# Oxymatrine Downregulates TLR4, TLR2, MyD88, and NF-*κ*B and Protects Rat Brains against Focal Ischemia

**DOI:** 10.1155/2009/704706

**Published:** 2010-02-16

**Authors:** Hongguang Fan, Litao Li, Xiangjian Zhang, Ying Liu, Chenhui Yang, Yi Yang, Jing Yin

**Affiliations:** Department of Neurology, Second Hospital of Hebei Medical University, Shijiazhuang, Hebei 050000, China

## Abstract

Inflammatory damage plays an important role in cerebral ischemic pathogenesis and may represent a target for treatment. Toll-like receptor-4 (TLR4), toll-like receptor-2 (TLR2), myeloid differentiation factor 88 (MyD88), and nuclear factor kappa-B (NF-*κ*B) have been linked to inflammatory reactions. Our previous studies have proved that oxymatrine (OMT) protected ischemic brain injury and this effect may be through the decreasing of NF-*κ*B expression. However, little is known regarding the mechanism of OMT in the acute phase of ischemic stroke. We therefore investigated the OMT's potential neuroprotective role and the underlying mechanisms. Male, Sprague-Dawley rats were randomly divided into sham, saline and OMT treatment groups. We used a middle cerebral artery occlusion (MCAO) model and administered OMT intraperitoneally immediately after cerebral ischemia and once daily on the following days. At time points after MCAO, brain water content and infarct size were measured. Immunohistochemistry and RT-PCR were used to analyse the expression of TLR4, TLR2, MyD88, and NF-*κ*B at gene and protein level in ischemic brain tissue. The result indicated that OMT protected the brain from damage caused by MCAO; this effect may be through downregulation of the TLR4, TLR2, MyD88, and NF-*κ*B.

## 1. Introduction

The excessive inflammatory immune reaction often resides in region of necrosis and ischemic tissue after cerebral infarction and leads to inflammatory injury. The relationship between toll-like receptor-4 (TLR4), toll-like receptor-2 (TLR2), and nonbiological inflammatory injury has been proved [1–3]. It is believed that injured tissue and necrotic cells release endogenous activators (adjuvants). These activators can combine with TLR4 in the cell membrane. With the assistance of cluster of differentiation-14 (CD14) and myeloid differentia protein 2 (MD2), they activate nuclear factor kappa-B (NF-*κ*B) and release a series of cytokines to counteract inflammation. Potential ligands also activate TLR2. They can also induce specific immunity to increase the self-protection ability of the organism. An excessive inflammatory reaction can also damage target cells and tissue [4, 5]. Reducing the inflammatory injury is therefore regarded to be one of major ways to treat acute cerebral infarction. 

The pathway mediated by TLR4 includes myeloid differentiation protein-88- (MyD88-) dependent and MyD88-independent pathways [6]. Through TLR4 intrinsic activator, the former can combine with CD14 and anchor on cell surface. Through the effect of MD2, the combination of intrinsic activator and TLR4 can induce a series of intracellular signal transductions. NF-*κ*B is activated to cause release of inflammatory factors. The MyD88-independent pathway belongs to the TLR4-TRIF (TIR-domain-containing adaptor protein inducing IFN*β*) pathway [7, 8] and also has a close relationship with TLR4. The pathway induced by TLR2 is restricted to MyD88-dependent pathways.

**Figure 1:**
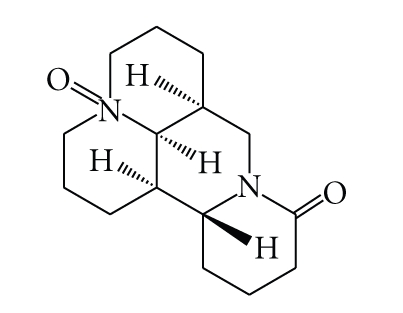
The chemical structure of OMT.

Oxymatrine (OMT) has a tetracyclic quinolizine structure. Its molecular formula is C_15_H_24_N_2_O. The structure of OMT is shown in Figure 1. It is an alkaloid extracted from *Sophora flavescens Ait* (a traditional Chinese medicine (TCM) belonging to the pulse family). The antiinflammatory, antioxidative and antivirus effects of OMT, as well as its role in immunological regulation, have been reported [9, 10]. In recent years, OMT studies have focused mainly on its therapeutic effect against hepatitis and certain types of tumor. There have been few reports on the influence of OMT on cerebrovascular disease. 

## 2. Materials and Methods

OMT was purchased from Shanxi Huike Botanical Development Company Limited (Shanxi, China). It had a purity of 98%. The fine powder of OMT was prepared as a suspension (40 mg/mL) with distilled water and stored at 4°C.

Animal resource and model building. Male Sprague-Dawley (SD) rats (240–260 g) were provided by the Experimental Animal Center, Preclinical Medical College, Hebei Medical University (Hebei, China). They were housed in multilayer laminar flow racks. They could move about freely and had a standard diet and purified water. The room temperature was maintained at 20–25°C. According to the methods reported by Longa [11] and Laing et al. [12], an animal model of middle cerebral artery occlusion (MCAO) was established by the suture embolic method.

### 2.1. Experimental Design

To observe the influence of OMT on infarction size, water content, and infiltration of inflammatory cells gene expression and protein expression of TLR4, TLR2, MyD88, and NF-*κ*B in ischemic brain tissue, rats were divided into three groups, that is, OMT treatment group (120 mg/kg), saline group, and sham operation group. Each group was divided into three subgroups at different time points (6 hours, 24 hours, and 48 hours after operation). OMT suspension (40 mg/mL) was given to rats in the OMT treatment group via intraperitoneal injection (120 mg/kg) after MCAO, then once a day. Rats in the saline group received 1 mL of saline replacing OMT after MCAO using the same method.

### 2.2. Detection of Water Content in Brain Tissue

The water content of brain tissue was detected using the dry-wet weight technique. Five rats of each group were killed, brain tissues were harvested quickly, and the frontal pole (thickness, 4 mm) removed. Brain tissue behind the frontal pole (thickness, 2 mm) was selected for detection of water content. After ascertaining the wet weight (WW) with an electronic balance, brain tissue was put into an oven at constant temperature (100 ± 5°C) for 24 hours. The dry weight (DW) was then obtained. Water content was calculated based on the formula: (WW − DW)/WW × 100%.

### 2.3. Determination of Infarction Size

2, 3, 5-triphenyltetrazolium (TTC) stain of brain tissue was used. Three rats of each group were killed, brain tissues were harvested quickly, and the frontal pole and cerebella removed. Coronal slices of thickness 2 mm were prepared. Brain tissue was divided into five portions, immersed in 2% TTC phosphonic buffer, and incubated at 37°C for 30 minutes. Viable brain tissue was stained red by TTC, whereas necrotic brain tissue was pale white. Slices were preserved in 10% formaldehyde. The apical side of each slice was imaged. The area of infarction on both sides of each slice was determined by an image analyzer.

### 2.4. Detection of Expression of TLR4, TLR2, MyD88, and NF-*κ*B in Brain Tissue by Immunohistochemistry

Sections were deparaffinized by a standard method. They were then incubated in 3% H_2_O_2_/methanol to eliminate endogenous peroxidase activity. After rinsed with phosphate-buffered saline (PBS; 0.01 M, pH 7.4) for antigen retrieval, sections were placed in citrate buffer (0.01 mol/L, pH 6.0) for 30 minutes. Sections were cooled at room temperature and incubated with normal goat serum. Sections were incubated successively with primary antibody (for TLR4 and NF-*κ*B, 1 : 400, Santa Cruz Biotechnology; for TLR2, 1 :  400, LifeSpan Bioscience; for MyD88, 1 : 100, Santa Cruz Biotechnology) overnight at 4°C. They were rinsed with PBS and incubated with biotinylation secondary antibody fluid at 37°C for 45 minutes. They were rinsed again with PBS and incubated with horseradish peroxidase-labeled streptoavidin at 37°C. Slices were developed with diaminobenzidene (DAB) and counterstained with hematoxylin. They were dehydrated and mounted. The expression of TLR4, TLR2, MyD88, and NF-*κ*B in brain tissue was calculated. Three sections of each brain were selected every 100 *μ*m interval. Six scopes were selected randomly in the infarcted cortex and immunopositive cells were counted. The average number was calculated and represented the immunopositive cells of that rat, and three rats were used in each group.

### 2.5. Reverse Transcription-Polymerase Chain Reaction (RT-PCR)

Ischemic brain tissue was harvested quickly. Total RNA was extracted with Trizol (Invitrogen) and the density and purity detected. RNA (2 *μ*g) of each sample was used for synthesizing cDNA through inverse transcription; 1 *μ*L of cDNA was used to carry out PCR amplification. Primers were synthesized by Shanghai Sangon Biological Engineering Technology Company Limited. Correctness of the gene order was proved in GenBank (Table 1). PCR conditions were initial denaturation for 2 minutes at 95°C, 35 cycles of amplification with denaturation at 95°C for 30 seconds, annealing at 52°C for 30 seconds, and extension at 72°C for 40 seconds. After 35 cycles, extension at 72°C for 5 minutes was done. RT-PCR products (5 *μ*L) were analyzed by 2% agarose gel electrophoresis. The gray scale of the electrophoresis strip was scanned by an ultraviolet photometry (UVP) gel imaging system. The relative expression of products was represented with TLR4/*β*-actin, TLR2/*β*-actin, MyD88/*β*-actin, and NF-*κ*B/*β*-actin; data were analyzed with an image analysis system.

### 2.6. Data Analysis

Data were analyzed using software SAS 6.8. Results were means ± SD. Single-factor variance analysis was used to compare the difference between groups, and the *q* test was used to compare differences between two groups. *P* < .05 was considered significant.

## 3. Results

### 3.1. OMT Reduced the Infarction Size of Ischemic Brain Tissue

**Figure 2:**
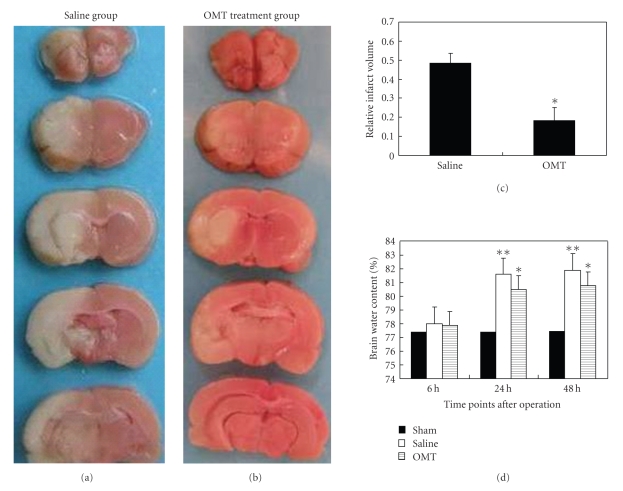
Relative infarct volume and water content in the brain. (a) and (b) TTC stain: comparison of infarct size between the saline group and OMT treatment group at 24 hours. The white region was the infarction and the red region was normal brain tissue. (c) Relative infarct volume: calculating and comparing the percentage of infarct region occupying the whole brain tissue area in one section revealed that it decreased by 32% in the OMT treatment group compared with the saline group at 24 hours. *P < .05 compared with saline group. (d) Water content of cerebral tissue: there was no difference at 6 hours among the test groups. It increased at 24 hours in the saline group and lasted up to 48 hours in the saline group at identical time points. Water content in the OMT treatment group decreased at 24 hours and 48 hours after the operation compared with that in the saline group at the same time points. Results are means ± SD of each group. **P < .01 and *P < .05 compared with Sham.

The infarction size in ischemic brain tissue was reduced by 32% in the OMT treatment group compared with that in the saline group 24 hours after operation (Figures 2(a), 2(b), and 2(c)).

### 3.2. OMT Reduced the Water Content of Brain Tissue

With respect to cerebral water content, the following results were noted. There was no difference at 6 hours among the test groups. No change was found at all time points in the sham group. Cerebral water content increased at 24 hours in the saline group and lasted up to 48 hours in the saline group. It was evidently lower in the OMT treatment group, and the difference was significant (Figure 2(d)).

### 3.3. OMT Reduced the Expression of TLR4, TLR2, MyD88, and NF-*κ*B mRNA

**Figure 3:**
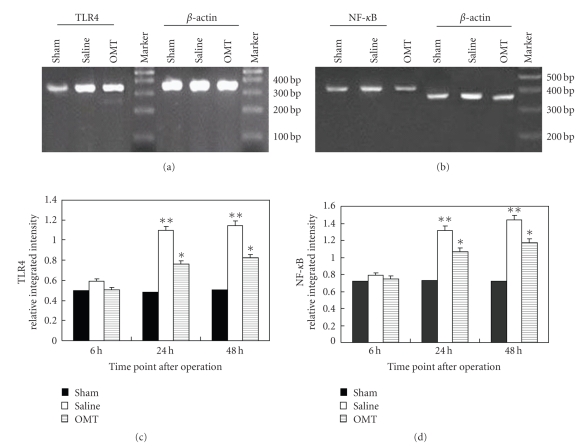
RT-PCR results of TLR4 and NF-*κ*B. (a) RT-PCR of TLR4 at 24 hours after the operation in each group: expression of TLR4 mRNA was evidently higher in the saline group compared with the OMT treatment group, and the difference was significant (*P* < .05). (b) RT-PCR of NF-*κ*B 24 hours after the operation in each group: expression of NF-*κ*B mRNA was similar to that of TLR4 mRNA. (c) RT-PCR of TLR4 at all time points: expression of TLR4 mRNA showed no difference among all test groups at 6 hours. It was evidently higher in the saline group at 12 hours and lasted up to 48 hours. Compared with the saline group at identical time points, expression of TLR4 mRNA was lower in the OMT treatment group, and the difference was significant **P < .01 and *P < .05 compared with Sham. (d) RT-PCR of NF-*κ*B at all time points: expression of NF-*κ*B mRNA was similar to that of TLR4 mRNA  **P < .01 and *P < .05 compared with Sham.

The expression of TLR4, TLR2, MyD88, and NF-*κ*B mRNA in the brain tissue of each group was detected by RT-PCR 24 hours after the operation. The level of TLR4 mRNA expression was higher in the saline group than that in the sham operation group, and was lower in the OMT treatment group than in the saline group. The differences had statistical significance (Figure 3(a)). Expression of NF-*κ*B mRNA 24 hours after the operation was higher in the saline group than in the sham group and was lower in the OMT treatment group than in the saline group. The differences had statistical significance (Figure 3(b)). Results are expressed as means ± SD of each group (**P < .01 and *P < .05 compared with Sham (Figures 3(c) and 3(d))).

**Figure 4:**
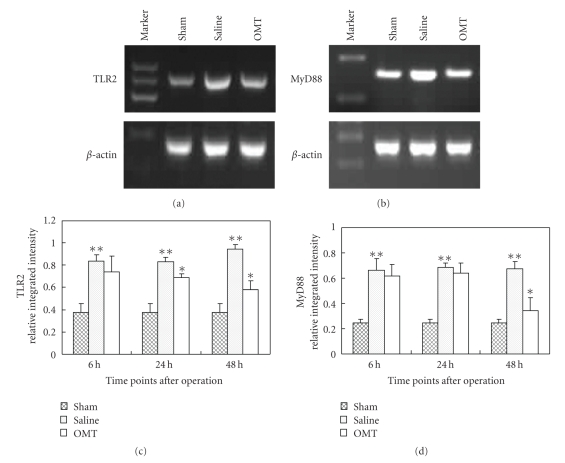
RT-PCR results of TLR2 and MyD88. (a) RT-PCR of TLR2 at 24 hours after the operation in each group: expression of TLR2 mRNA was evidently higher in the saline group compared with that in the OMT treatment group, and the difference was significant (*P* < .05). (b) RT-PCR of MyD88 at 48 hours after the operation in each group: expression of MyD88 mRNA was similar to that of TLR2 mRNA. (c) RT-PCR of TLR2 at all time points: expression of TLR2 mRNA was evidently higher in the saline group at 6 hours and lasted up to 48 hours. There was no difference between saline and OMT group at 6 hours. Compared with the saline group at 24 hours and 48 hours, expression of TLR2 mRNA was lower in the OMT treatment group, and the difference was significant **P < .05 compared with Sham and *P < .05 compared with saline group. (d) RT-PCR of MyD88 at all time points: expression of mRNA was similar to that of TLR2 mRNA. **P < .05 compared with Sham and *P < .05 compared with saline group.

The level of TLR2 mRNA expression was higher in the saline group than that in the sham operation group and was lower in the OMT treatment group than in the saline group. The differences had statistical significance (Figure 4(a)). Expression of MyD88 mRNA 48 hours after the operation was higher in the saline group than in the sham group and was lower in the OMT treatment group than in the saline group. The differences had statistical significance (Figure 4 part (b)). Results are expressed as means ± SD of each group (**P < .05 compared with Sham and *P < .05 compared with saline group (Figures 4(c) and 4(d))).

### 3.4. OMT Reduced the Expression of TLR4, TLR2, MyD88, and NF-*κ*B Protein

**Figure 5:**
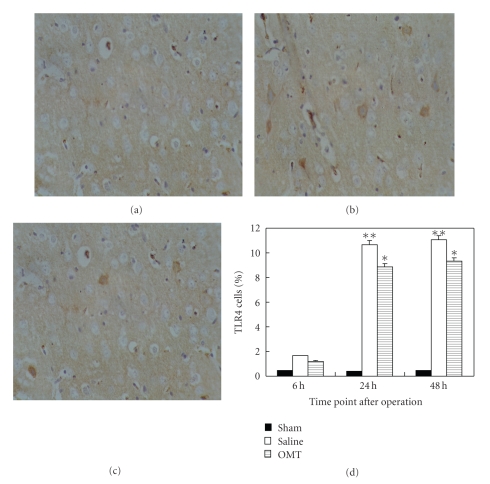
TLR4 expression detected by immunohistochemistry. (a) TLR4 expression in the sham group: TLR4 expression could not be observed in the sham group at 24 hours. (b) TLR4 expression in the saline group: TLR4 expression evidently increased in the saline group at 24 hours. Positive cells were defined as having buffy grains in the cytoplasm. (c) TLR4 expression in the OMT treatment group: TLR4 expression decreased relatively in the OMT treatment group at 24 hours. (d) TLR4 expression in each group at all time points: TLR4 expression showed no difference among all the test groups at 6 hours. It was evidently higher in the saline group at 24 hours and lasted up to 48 hours. Compared with the saline group at identical time points, TLR4 expression was lower in the OMT treatment group, and the difference was significant **P < .01 and *P < .05 compared with Sham.

Expression of TLR4 protein in cerebral tissue was detected by an immunohistochemical technique at 24 hours after the operation in each group. TLR4 expression in the sham group is shown in Figure 5(a). A high level of TLR4 expression in the saline group was noted (Figure 5(b)). TLR4 expression was lower in the OMT treatment group than in the saline group; the difference had statistical significance. Positive cells were defined as having buffy grains in the cytoplasm (Figure 5(c)). Results are expressed as means ± SD of each group (**P < .01 and *P < .05 compared with Sham (Figure 5(d))).

**Figure 6:**
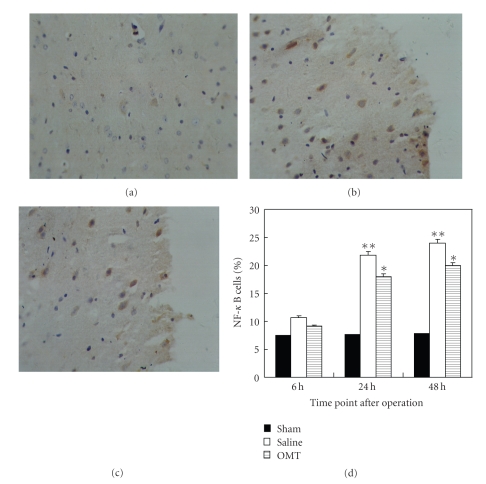
NF-*κ*B expression detected by immunohistochemistry. (a) NF-*κ*B expression in the sham group: NF-*κ*B expression could be observed periodically in the sham group at 24 hours. (b) NF-*κ*B expression in the saline group: NF-*κ*B expression evidently increased in the saline group at 24 hours. Positive cells were defined as haning buffy grains in the cellular nucleus and cytoplasm. (c) NF-*κ*B expression in the OMT treatment group: NF-*κ*B expression decreased relatively in the OMT treatment group at 24 hours. (d) NF-*κ*B expression in each group at all time points: there was no difference among all test groups at 6 hours. It was evidently higher in the saline group at 24 hours and lasted up to 48 hours. Compared with the saline group at identical time points, NF-*κ*B expression was lower in the OMT treatment group, and the difference was significant **P < .01 and *P < .05 compared with Sham.

Expression of NF-*κ*B protein 24 hours after the operation in each group was noted using an immunohistochemical technique. NF-*κ*B expression in the sham group is shown in Figure 6(a). A high level of NF-*κ*B expression in the saline group was observed (Figure 6(b)). NF-*κ*B expression was lower in the OMT group than in the saline group, and the difference had statistical significance. Positive cells were defined as having buffy grains in the nucleus and cytoplasm (Figure 6(c)). Results are expressed as means ± SD of each group (**P < .01 and *P < .05 compared with Sham (Figure 6(d))).

**Figure 7:**
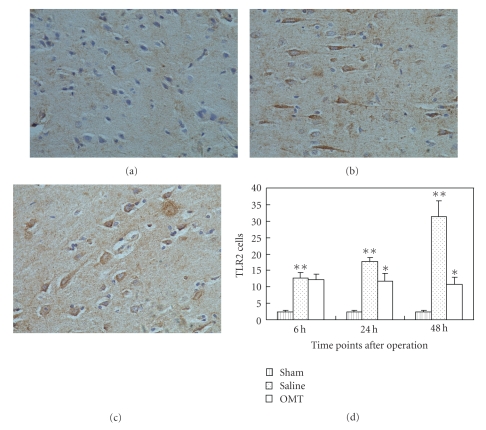
TLR2 expression detected by immunohistochemistry. (a) TLR2 expression in the sham group: TLR2 expression could be observed periodically in the sham group at 24 hours. (b) TLR2 expression in the saline group: TLR2 expression evidently increased in the saline group at 24 hours. Positive cells were defined as presenting buffy grains in cytoplasm. (c) TLR2 expression in the OMT treatment group: TLR2 expression decreased relatively in the OMT treatment group at 24 hours. (d) TLR2 expression in each group at all time points: expression of TLR2 protein was evidently higher in the saline group at 6 hours and lasted up to 48 hours. There was no difference between saline and OMT group at 6 hours. Compared with the saline group at 24 hours and 48 hours, expression of TLR2 protein was lower in the OMT treatment group, and the difference was significant **P < .05 compared with Sham and *P < .05 compared with saline group.

Expression of TLR2 protein 24 hours after the operation in each group was noted using an immunohistochemical technique. TLR2 expression in the sham group is shown in Figure 7(a). A high level of TLR2 expression in the saline group was observed (Figure 7(b)). TLR2 expression was lower in the OMT group than in the saline group, and the difference had statistical significance. Positive cells were defined as having buffy grains in the cytoplasm (Figure 7(c)). Results are expressed as means ± SD of each group (**P < .05 compared with Sham and *P < .05 compared with saline group (Figure 7(d))).

**Figure 8:**
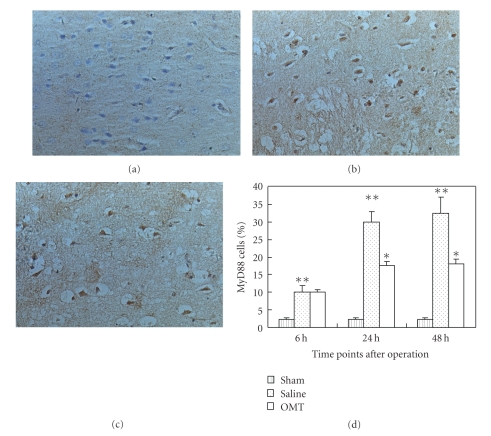
MyD88 expression detected by immunohistochemistry. (a) MyD88 expression in the sham group: MyD88 expression could be observed periodically in the sham group at 24 hours. (b) MyD88 expression in the saline group: MyD88 expression evidently increased in the saline group at 24 hours. Positive cells were defined as presenting buffy grains in cytoplasm. (c) MyD88 expression in the OMT treatment group: MyD88 expression decreased relatively in the OMT treatment group at 24 hours. (d) MyD88 expression in each group at all time points: Expression of MyD88 protein was evidently higher in the saline group at 6 hours and lasted up to 48 hours. There was no difference between saline and OMT group at 6 hours. Compared with the saline group at 24 hours and 48 hours, expression of MyD88 protein was lower in the OMT treatment group, and the difference was significant **P < .05 compared with Sham and *P < .05 compared with saline group.

Expression of MyD88 protein 24 hours after the operation in each group was noted using an immunohistochemical technique. MyD88 expression in the sham group is shown in Figure 8(a). A high level of MyD88 expression in the saline group was observed (Figure 8(b)). MyD88 expression was lower in the OMT group than in the saline group, and the difference had statistical significance. Positive cells were defined as having buffy grains in the cytoplasm (Figure 8(c)). Results are expressed as means ± SD of each group (**P < .05 compared with Sham and *P < .05 compared with saline group (Figure 8(d))).

## 4. Discussion

The antiinflammatory and antioxidative effects of OMT (as well as its roles in immunological regulation) have attracted attention over the past few years [13, 14]. It has been confirmed that OMT can act on nonspecific inflammatory reactions [15]. OMT exerts its antiinflammatory effect without dependence of the pituitary-adrenal system: it acts directly on inflammatory cells [15]. OMT is a two-way immunoregulant. That is, it can stimulate the proliferation of lymphocytes at low concentrations, and yet inhibit this proliferation at high concentrations. 

Cerebral infarction can be observed in multiple pathological processes [16] and can be affected by multiple factors. In general, it is believed that cerebral infarction is a typical noninfective lesion, and that its pathophysiological mechanism involves the production of oxygen free radicals and release of inflammatory mediators. TLRs are thought to have important effects on nonbiological inflammatory lesions [1, 17].

TLRs are type-I transmembrane receptors. Their extracellular domain is a pathogen-associated molecular pattern (PAMP). Their intracellular part is a structural domain named Toll/IL-1R (TIR), which can conduct stimulating signals. At least 11 TLRs (TLR1–TLR11) have been identified. TLR4 and TLR2 have been confirmed to participate in the inflammatory reaction, and to promote the maturation and differentiation of immune cells. Various TLRs are expressed in human and murine brains [18, 19]. TLR4 is extensively expressed in the periventricular plexus, microglial cells and astrocytes. TLR4 mRNA is also observed in intracerebral vascular smooth muscle cells and epithelia. TLR2 has been described to express in astrocytes, microglia, endothelial cells, ependymal cells, as well as neurons. Many PAMPs can activate TLR4, including peptidoglycan (PGN), Lipoteichoic acid (LTA), lipoprotein, and oxidative stress (i.e., oxygen free radicals). Potential ligands for TLR2 activation are some endogenous “danger molecules” like high mobility group box protein 1 (Hmgb1) or heat shock protein 70 (HSP70) [20, 21]. Activated TLR4 and TLR2 then transfer signals to NF-*κ*B through MyD88-dependent and independent pathways and induce activation of proinflammatory cytokines such as tumor necrosis factor-alpha (TNF-*α*) and interleukins (ILs). TLR family members recognize not only exogenous microorganisms but also endogenous harmful substances [22]. For example, they are involved in the responses to necrotized cells, heat-shock protein, and the degradation products of the extracellular matrix. It was recently indicated that TLR4 and TLR2 have critical effects in injuries to the central nervous system [23–25] and that the microglial cell is the main mediator of such injuries. As an inflammatory transmembrane receptor, the role of TLRs has become a controversial issue in the field of infective diseases. There are many studies on the effects of TLR4 in nonbiological inflammatory injuries (e.g., ischemia of the myocardium, liver, and brain), but there are few reports on ischemic nonbiological infective injury. After cerebral infarction, it has been confirmed that the infective injury around ischemic tissue becomes the direct cause of cerebral edema [1] but does infective injury after cerebral infarction correlate to TLR4 and TLR2 levels? 

We calculated the water content of brain tissue and the infarction size (as determined by TTC stain) to evaluate the influence of OMT on the expression of TLR4, TLR2, MyD88 and NF-*κ*B in focal cerebral infarction in rats. Expression of TLR4, TLR2, MyD88, and NF-*κ*B mRNA and protein in test groups was detected by RT-PCR and immunohistochemistry, respectively. OMT could reduce water content and reduce the infarction area of ischemic brain tissue. It could also weaken the expression of TLR4, TLR2, MyD88, and NF-*κ*B mRNA and protein. We hypothesized that after endogenous activators were identified by TLR4 or TLR2, the signal transduction pathway was switched on. This led to recruitment of MyD88, activation of NF-*κ*B and the subsequent release of proinflammatory cytokines, resulting in tissue injury. By decreasing the expression of TLR4 and TLR2 gene and protein, OMT can reduce the expression of MyD88 and NF-*κ*B gene and protein. This can inhibit inflammatory injury, weaken cerebral edema, and provide neuroprotection against subsequent cerebral ischemic injury. How TLR4, TLR2, and a serial of following inflammatory factors (such as MyD88 and NF-*κ*B) regulate the immune system require further research. Our study showed that administration of 120 mg/kg OMT was sufficient to provide significant neuroprotection against neurological injury induced by MCAO. We supported this finding by both histological and neurological data: a reduction in the brain infarct volume. These results showed that OMT had beneficial effects for ischemia. However, the potential mechanisms underlying the neuroprotection of OMT are not yet known. Our study showed that OMT may ameliorate the inflammation through reducing the expression of TLR4, TLR2, MyD88, and NF-*κ*B.

In conclusion, our study has confirmed that OMT protected the brain from damage caused by MCAO; this effect may be through downregulating the expression of TLR4, TLR2, MyD88, and NF-*κ*B. The elevated expression of TLR4, TLR2, MyD88, and NF-*κ*B is thought to be consistent with ischemia evoked neuron injury and death through inflammatory mechanism. TLR4, TLR2, MyD88, and NF-*κ*B pathway may be one of the strategic targets for cerebral ischemic therapies and OMT maybe a novel, effective therapeutic drug for the treatment of ischemic brain injury.

## Figures and Tables

**Table 1:** Summary of the RT-PCR Primers sequences.

Gene	Primers	Sequences
TLR4	Forward	5′-GCC GGA AAG TTA TTG TGG TGG T-3′
	Reverse	5′-ATG GGT TTT AGG CGC AGA GTT T- 3′

TLR2	Forward	5′-GAA AGA TGC GCT TCC TGA AC-3′
	Reverse	5′-CGC CTA AGA GCA GGA TCA AC-3′

MyD88	Forward	5′-CAA CCA GCA GAA ACA GGA GTC T-3′
	Reverse	5′-ATT GGG GCA GTA GCA GAT GAA G-3′

NF-*κ*B	Forward	5′-GCG CAT CCA GAC CAA CAA TAA C-3′
	Reverse	5′-GCC GAA GCT GCA TGG ACA CT- 3′

*β*-actin	Forward	5′-GCC ATG TAC GTA GCC ATC CA-3′
	Reverse	5′-GAA CCG CTC ATT GCC GAT AG -3′
